# History of Incarceration is Associated with Unmet Socioeconomic Needs and Structural Discrimination among Young Black Sexual Minority Men (SMM) in the United States

**DOI:** 10.1007/s11524-023-00737-8

**Published:** 2023-05-19

**Authors:** Adedotun Ogunbajo, Daniel Siconolfi, Erik Storholm, Wilson Vincent, Lance Pollack, Greg Rebchook, Judy Tan, David Huebner, Susan Kegeles

**Affiliations:** 1 RAND Corporation, 1776 Main St, Santa Monica, CA 90401 USA; 2 San Diego State University, School of Public Health, San Diego, CA USA; 3 Department of Psychology, Temple University, Philadelphia, PA USA; 4 University of California, San Francisco, School of Medicine, San Francisco, CA USA; 5 George Washington University, Milken School of Public Health, Washington, DC USA

## Abstract

There is a dearth of research on incarceration among young Black sexual minority men (SMM). The current study aimed to assess the prevalence and association between unmet socioeconomic and structural needs and history of incarceration among young Black SMM. Between 2009 and 2015, young Black SMM (N = 1,774) in Dallas and Houston Texas were recruited to participate in an annual, venue-based, cross-sectional survey. We found that 26% of the sample reported any lifetime history of incarceration. Additionally, participants with unmet socioeconomic and structural needs (unemployment, homelessness, financial insecurity and limited educational attainment) were more likely to have a history of incarceration. It is imperative that interventions are developed to address the basic, social, and economic needs of young Black SMM with a history of incarceration or who are at risk for incarceration.

## Introduction

In the United States (U.S.), racial disparities in the criminal justice system have been widely documented. Black Americans have higher rates of arrests, convictions, and sentencing, compared to White Americans [1]. Furthermore, the lifetime risk of incarceration for a Black man born today is 1 in 3, compared to 1 in 6 among Latino men and 1 in 17 among White men [1]. These disparities are rooted in the racist foundation which continues to be perpetuated through the current criminal system and prison industrial complex in the U.S. [2]. Historians have drawn a through line from the utilization of jails and prisons to reinforce the institution of slavery in the Southern U.S. to the use of police forces to quell the civil right movement protests in the 1960s, to the war on drugs and gangs in the 1980s and 1990s to the police brutality cases that precipitated the present contemporary Black Lives Matter movement [3].

While the national discourse on criminal justice has centered the disproportionate injustices experienced by racial and ethnic minority groups, sexual and gender minority groups (including sexual and gender minority persons who also have minoritized racial or ethnic identities) have received less attention. However, studies have shown that sexual minority communities experience high levels of involvement with the criminal justice system. For example, a national study found that the incarceration rate of individuals who identify as lesbian, gay, or bisexual was three times that of the general U.S. population, and these communities were more likely to be sexually victimized while incarcerated, experience solitary confinement, and have psychological distress [4].

However, the focus on a singular axis of oppression and social marginalization (i.e., race, sexual orientation, gender etc.) erases the lived experiences of people who exist at the intersection of multiple marginalized and minoritized identities. Intersectionality theory asserts that identities are not merely independent and additive but interlocking and mutually constitutive [5, 6]. Specifically, Black sexual minority men (SMM) exist at the intersection of racism, homophobia, and heterosexism, which might significantly increase their chances of coming in contact with the U.S. criminal justice system. For example, a national study of Black SMM in 6 cities across the U.S. found that 61% reported being incarcerated at least once in their lifetime [7]. Another study of Black SMM in the Deep South (Georgia and Mississippi) found that 36% of participant had a history of incarceration [8]. Additionally, individuals with a history of incarceration may have limited economic opportunities, such as barriers to employment, which may contribute to recidivism. Various studies have documented that SMM with a history of incarceration are more likely to report lower income, engage in transactional sex (sex in exchange for money, food, housing, etc.) and substance use [9–12]. These limited data suggest that Black SMM might be disproportionally represented in the criminal justice system, which has implications for the health and wellbeing of this marginalized group.

Many of the negative impacts of incarceration are downstream effects attributed to stress, stigma, and limited socio-economic resources and opportunities [13–16] (i.e., negative impacts on education, housing, employment, financial security, health care access). Past studies of incarceration among SMM have been within the context of HIV, specifically as an indicator of underlying risk for HIV seroconversion or lapses in the HIV care continuum. For example, a study of young SMM in Chicago, IL found that 41% had a prior history of criminal justice involvement, 35% were living with HIV, and among those living with HIV, having a history of criminal justice involvement was associated with better HIV-related health outcomes compared to those with no prior history of criminal justice involvement [17]. Another study found no association between incarceration and new cases of HIV among a cohort of Black SMM [18]. While these studies have contributed to our knowledge base on the relationship between incarceration and HIV, we also recognize that Black SMM are confronted with other challenges beyond HIV.

There is a dearth of research on the non-HIV health impacts of incarceration on Black SMM. Recent qualitative research with young Black SMM found that incarceration negatively impacted employment and economic security [19]. Another study found that economic hardship, prior incarceration, and substance use were associated with incident incarceration among young Black SMM and transgender women[20]. More research is needed on the relationship between incarceration and the re-entry needs among sexual and gender minorities, specifically employment, housing, and access to health care among Black SMM with a history of incarceration [14, 21, 22].

To address this research gap, we conducted secondary analysis of data from a large, multi-year survey of young Black SMM in Houston and Dallas, Texas. The objective of the current work was to assess the association between unmet socioeconomic needs (i.e., employment, housing, healthcare, etc.), structural discrimination (i.e., homophobia and racism) and history of incarceration among young Black SMM in the U.S. South. We hypothesized that having a history of incarceration would be associated with higher current socioeconomic and structural needs. This analysis will help provide additional insights into the potential services and programs that may be needed to assist young Black SMM after release from correctional facilities or those at risk for incarceration.

## Methods

### Participants and Procedures

Between 2009 and 2015, young Black SMM in Dallas and Houston Texas were recruited to participate in an annual, venue-based, cross-sectional survey. The majority of the sample (> 90%) were recruited at bars and clubs frequented by SMM. The current study includes men who participated in cohorts 5 (2013), 6 (2014), and 7 (2015). The survey took about 30 minutes to complete, and participants were reimbursed $30 for their time. All data were self reported. To facilitate identification of duplicate participants across the three study waves, several time-invariant personal characteristics (e.g., the first letter of their mother’s name) were checked. While performing data merging procedures, each participant was assigned a unique alphanumeric identifier constructed by combining those characteristics. We retained the first observation for participants who completed the surveys at multiple waves after removing duplicates (N = 1774). More details on the study methodology have been published elsewhere in publicly available manuscripts [23–26].

### Measures

#### Sociodemographic Characteristics

Participants reported their age and location (Houston or Dallas, Texas). For analysis, participants’ educational level was categorized as having completed high school/GED versus not having completed high school/GED. HIV status was coded as HIV-negative, HIV-positive, or unsure/unknown serostatus for analysis. Sexual orientation was measured as *gay, homosexual, same gender loving, *etc*.; bisexual; heterosexual or straight; other;* the latter two categories were collapsed for analysis based on cell sizes.

#### Socioeconomic Needs

We collected information about current employment status, history of homelessness, history of exchange sex in the previous 60 days (a marker of sexual behavior due to socioeconomic disadvantage and need), whether the participant had run out of money during one or more months in the previous year, whether the participant needed to borrow money in the previous year, whether the participant had a primary care provider, and history of testing for an STD in previous year (having a primary care provider and history of STD testing were operationalized as proxy variables for access to healthcare services, which is highly determined by socioeconomic status).

### Structural Discrimination

#### Experienced Racism

We measured experienced racism using an 11-item scale developed by Díaz et al. (2004) [27]. Items assessed a range of potential experiences in the past year (e.g., “In the past year, how often have you heard (or been told) a racially offensive comment or joke?” and “In the past year, how often have your civil rights been violated (i.e., job or housing discrimination due to racism, racial discrimination, or racial prejudice)?). Participants responded using a 5-point scale ranging from *never* to *very often*, and items were summed to create a total score, where a higher score indicates greater experiences of racism in the past year.

#### Experienced Homophobia

We measured experienced homophobia using a 7-item scale developed by Díaz et al. (2004) [27]. Items assessed a range of potential experiences in the past year (e.g., “In the past year, how often have you had to pretend that you're totally straight or heterosexual in order to be accepted?” and “In the past year, how often were you made fun of or called names for being effeminate (“girly”) or for being attracted to other men (or gay or bisexual)?”). Participants responded using a 5-point scale ranging from *never* to *very often*, and items were summed to create a total score, where a higher score indicates greater experiences of homophobia in the past year.

#### Mental Health

We utilized a shortened, 8-item version of the Center for Epidemiologic Studies Depression Scale (CES-D)[28] scale to measure depressive symptoms. Responses are summed and a higher score indicates greater depressive symptoms.

#### Resilience

We utilized the Wagnild and Young 14-item psychological resilience scale [29]to measure resilience. Participants responded to items (e.g., “I usually manage one way or another”) using a six-point Likert scale from *disagree strongly* to *agree strongly*. Greater scores indicate greater psychological resilience.

#### Substance Use

To assess binge drinking, participants were asked “During the past 60 days, on how many days did you have 5 or more drinks on the same occasion?” and we created a *none* versus *any* dichotomous variable for analysis. Stimulant use was also measured with a 60-day recall period; for analysis, we combined responses for ecstasy/MDMA, crack, cocaine, and methamphetamine to create a single *none* versus *any* stimulant use variable. Smoking status was derived from an item asking the number of days in the past 60 days that the respondent had smoked cigarettes. From this, we created a trichotomous variable (non-smoker, 0 days; non-daily smoker, 1–59 days; daily smoker, 60 days).

#### History of Incarceration

Participants were asked, “Were you ever in juvenile, jail, or prison?” and response categories included *No, I have never been in juvenile, jail, or prison*; *Yes, in the last 2 months*; *Yes, more than 2 months ago but within the last year*; *Yes, more than a year ago*. Respondents were only allowed to choose one answer, and the question did not instruct participants how to respond if they had experienced multiple incarcerations (e.g., in reporting the recency, some men may have referenced their first incarceration experience, while others may have referenced their most recent experience). Given this lack of specificity and the ambiguity it would introduce to the interpretation of results, we created a collapsed dichotomous variable reflecting *any lifetime incarceration experience*.

### Data Analysis

Variables that had theoretical and domain relevance to incarceration history based on the scientific literature were included for analysis. Bivariate and multivariable logistic regression models were constructed to examine the unadjusted and adjusted relationship between each variable and history of incarceration. Variables that had theoretical and domain relevance based on the scientific literature were retained in the multivariable models assessing these outcomes. We constructed four multivariable models to test incremental associations between the independent variables and any lifetime on history of incarceration. This included model #1 (Structural & socioeconomic needs only), model #2 (Structural, socioeconomic needs & mental health outcomes only), model #3 (Structural, socioeconomic needs, and substance use but no mental health outcomes) and the full model, model #4 (Structural, socioeconomic needs, substance use, and mental health outcomes). We utilized this forward stepwise approach to examine which factors remained significant or became insignificant with each addition of co-variates category to the model. Data was analyzed using Stata MP 17.

## Results

Sociodemographic, socioeconomic, and structural discrimination variables are presented in Table 1. Over a quarter (26%) of the sample had any lifetime experience of incarceration. The sample was evenly split between Dallas (51%) and Houston (49%). A majority of the sample identified as gay/homosexual/same gender loving (78%) and self-reported being HIV negative (78%). Only 15% reported not having a high school diploma or GED and 9% were out of work (unemployed, not a student, or on disability).

**Table 1:** Correlates of history of incarceration by sociodemographic characteristics, socioeconomic needs, structural discrimination, mental health, and substance use behavior among a sample of young Black sexual minority men in the United States (N = 1,774)

	History of incarceration			
	Overall sample (N = 1,774)	Never incarcerated (n = 1,307,73.7%)	Ever incarcerated (n = 467, 26.3%)	*p*
Ever incarcerated	26.3%			
Age (in years)	24.9(2.9)	24.9	24.7	0.10
City				0.18
Dallas	900 (50.7%)	676 (51.7%)	224 (48.0%)	
Houston	874 (49.3%)	631 (48.3%)	243 (52.0%)	
HIV Status				** < 0.001**
Negative	1383 (78.4)	1047 (80.4%)	336 (72.6%)	
Positive	232 (13.1)	166 (12.8%)	66 (14.3%)	
Unsure/unknown	150 (8.5%)	89 (6.8%)	61 (13.2%)	
Sexual Orientation				0.16
Gay/Homosexual/same gender loving	1380 (77.9%)	1032 (79.0%)	348 (74.8%)	
Bisexual	355 (20.1%)	250 (19.1%)	105 (20.1%)	
Heterosexual/other	36 (2.0%)	24 (1.8%)	12 (2.6%)	
Exchange Sex				
Yes	135 (8.4%)	83 (7.0%)	52 (12.3%)	** < 0.001**
No	1468 (91.6%)	1097 (93.0%)	371 (87.7%)	
Socioeconomic needs and structural discrimination				
No high school diploma or GED	255 (14.5%)	152(11.7%)	103 (22.4%)	** < 0.001**
Out of work (unemployed, not a student, or on disability)	163 (9.2%)	104 (8.0%)	59 (12.8%)	**0.002**
Homelessness in the past year	163 (9.2%)	85 (6.5%)	78 (16.7%)	** < 0.001**
Ran out of money, 1 + months in the past year	818 (46.7%)	548 (42.4%)	270 (59.0%)	** < 0.001**
Needed to borrowed money to get by in past year	669 (38.0%)	453 (34.8%)	216 (46.8%)	** < 0.001**
No primary care provider (if HIV + , no HIV PCP)	400 (24.5%)	275 (22.8%)	125 (29.3%)	**0.009**
Tested for STD in previous year	1,177 (66.9%)	898 (69.2%)	279 (60.4%)	** < 0.001**
Experienced racism – score (Range = 11 to 55)	M = 23.3 (SD = 10.4)	M = 22.3	M = 25.7	** < 0.001**
Experienced homophobia – score (Range = 7 to 14	M = 15.6 (SD = 6.7)	M = 15.1	M = 17.0	** < 0.001**
Mental Health and substance use characteristics				
Depressive symptoms– score (Range = 0 to 21)	M = 4.6 (SD = 5.0)	M = 4.4	M = 5.4	** < 0.001**
Resilience – score (Range = 14 to 84)	M = 73.5 (SD = 15.3)	M = 74.3	M = 71.7	0.002
Binge drank, past 2 months	1,114 (65.1%)	794 (62.5%)	320 (72.6%)	** < 0.001**
Stimulant use, past 2 months	427 (24.3%)	257 (19.8%)	170 (36.9%)	** < 0.001**
Current smoker	785 (44.4%)	508 (39.0%)	277 (59.7%)	** < 0.001**
Smoking Frequency				
Nonsmoker	983 (55.6%)	796 (61.0%)	187 (40.3%)	
Non-daily smoker	543 (30.7%)	358 (27.5%)	185 (39.9%)	
Daily smoker	242 (13.7%)	150 (22.5%)	92 (19.8%)	

^*^Bolded values are for *p* < 0.05

Almost half (47%) of the sample reported having run out of money in at least one month in the previous year, 38% reported needing to borrow money to get by in the previous year, and 9% reported having experienced homelessness in the previous year. A quarter reported having no long-term primary care provider and about two-thirds (67%) had tested for a sexually transmitted disease in the previous year. Nearly two-thirds (65%) reported binge drinking in the previous 2 months, 44% were current smokers, and 24% had used stimulants in the previous 2 months. Bivariate associations between sociodemographic characteristics, socioeconomic and structural needs, and history of incarceration are presented in Table 2.

**Table 2:** Bivariate model predicting history of incarceration by sociodemographic characteristics, socioeconomic needs, structural discrimination, mental health, and substance use behavior among a sample of young Black sexual minority men in the United States (N = 1,774)

	Unadjusted logistic regression model for history of incarceration	
	Odds Ratio (95% Confidence Interval)	*p*
Age (in years)	0.92 (0.82–1.02)	0.10
City		
Dallas	Ref	
Houston	1.16 (0.94–1.45)	0.16
HIV Status		
Negative	Ref	
Positive	1.24 (0.91–1.69)	0.18
Unsure/unknown	2.14 (1.51–3.03)***	** < 0.001**
Sexual Orientation		
Gay/Homosexual/same gender loving	Ref	
Bisexual	1.25 (0.96–1.61)	0.96
Heterosexual/other	1.48 (0.73–3.00)	0.37
Exchange Sex		
Yes	1.85 (1.29–2.67)***	** < 0.001**
No	Ref	
Socioeconomic needs and structural discrimination		
No high school diploma or GED		
Yes	2.19 (1.67–2.88)***	** < 0.001**
No	Ref	
Out of work (unemployed, not a student, or on disability)		
Yes	1.69 (1.21–2.37))**	**0.002**
No	Ref	
Homelessness in the past year		
Yes	2.88 (2.08–4.00)***	** < 0.001**
No	Ref	
Ran out of money, 1 + months in the past year		
Yes	1.95 (1.57–2.42)***	** < 0.001**
No	Ref	
Needed to borrowed money to get by in past year		
Yes	1.64 (1.33–2.04)***	** < 0.001**
No	Ref	
No primary care provider (if HIV + , no HIV PCP)		
Yes	1.40 (1.10–1.80)**	**0.007**
No	Ref	
Tested for STD in previous year		
Yes	0.68 (0.55–0.85)***	**0.001**
No	Ref	
Experienced racism	1.38 (1.24–1.53)***	** < 0.001**
Experienced homophobia	1.32 (1.19–1.47)***	** < 0.001**
Mental Health and substance use characteristics		
Depressive symptoms	1.20 (1.09–1.33)***	** < 0.001**
Resilience	0.86 (0.77–0.95)**	0.002
Binge drank, past 2 months		
Yes	1.59 (1.25–2.01)***	** < 0.001**
No	Ref	
Stimulant use, past 2 months		
Yes	2.36 (1.87–2.99)***	** < 0.001**
No	Ref	
Smoking Frequency		
Nonsmoker	Ref	
Non-daily smoker	2.20 (1.73–2.79)***	** < 0.001**
Daily smoker	2.61 (1.93–3.54)***	** < 0.001**

^*^p < 0.05

^**^p < 0.01

^***^p < 0.001

In the first adjusted regression model, history of incarceration was regressed onto socioeconomic needs and structural discrimination, and participants with a history of incarceration had increased odds of multiple correlates. These correlates included: being unsure of their HIV status (OR 1.83; 95% CI: 1.21–2.78), not having a high school diploma or GED (OR 1.90; 95% CI: 1.34–2.70), being out of work (OR 1.64; 95% CI: 1.13–2.38), having experienced homelessness in the previous year (OR 1.78; 95% CI: 1.18–2.68), and having run out of money for basic needs in the previous year (OR 1.68; 95% CI: 1.30–2.17). Participants with a history of incarceration had decreased odds of reporting having tested for a sexually transmitted disease in the previous year (OR 0.72; 95% CI: 0.56–0.94) Table 3.

**Table 3:** Multivariable models predicting history of incarceration by sociodemographic characteristics, socioeconomic needs, structural discrimination, mental health, and substance use behavior among a sample of young Black sexual minority men in the United States (N = 1,714)

	Adjusted logistic regression model for history of incarceration			
	Model #1 (Structural & socioeconomic needs only)	*Model #2 (Structural, socioeconomic needs & mental health outcomes)*	*Model #3 (Structural, socioeconomic needs, and substance use but no mental health outcomes)*	*Model #4 (Structural, socioeconomic needs, substance use, and mental health outcomes)*
Age (in years)	0.99 (0.88–1.12)	0.98 (0.86–1.11)	0.96 (0.84–1.09)	0.94 (0.83–1.07)
City				
Dallas	Ref	Ref	Ref	Ref
Houston	1.11 (0.86–1.42)	1.12 (0.87–1.45)	1.13 (0.87–1.47)	1.12 (0.87–1.44)
HIV Status				
Negative	Ref	Ref	Ref	Ref
Positive	1.18 (0.83–1.68)	1.17 (0.81–1.67)	1.16 (0.81–1.67)	1.17 (0.82–1.68)
Unsure/unknown	1.83 (1.21–2.78)**	1.73 (1.12–2.65)*	1.97 (1.28–3.04)**	1.87 (1.22–2.87)**
Sexual Orientation				
Gay/Homosexual/same gender loving	Ref	Ref	Ref	Ref
Bisexual	1.30 (0.97–1.75)	1.37 (1.08–1.85)*	1.26 (0.93–1.71)	1.23 (0.91–1.66)
Heterosexual/other	2.65 (1.16–6.05)	3.01 (1.30–6.96)**	2.38 (1.02–5.56)*	1.74 (0.78–3.87)
Exchange Sex				
Yes	Ref	Ref	Ref	
No	1.48 (0.96–2.26)	1.35 (0.87–2.08)	1.16 (0.73–1.83)	
Socioeconomic needs and structural discrimination				
No high school diploma or GED				
Yes	1.90 (1.34–2.70)***	2.00 (1.38–2.89)***	1.86 (1.28–2.71)**	1.74 (1.19–2.53)**
No	Ref	Ref	Ref	Ref
Out of work (unemployed, not a student, or on disability)				
Yes	1.64 (1.13–2.38)*	1.67 (1.14–2.45)**	1.61 (1.10–2.37)*	1.63 (1.11–2.39)*
No	Ref	Ref	Ref	Ref
Homelessness in the past year				
Yes	1.78 (1.18–2.68) ***	1.61 (1.06–2.46)**	1.54 (1.00–2.38)	1.50 (0.99–2.28)
No	Ref	Ref	Ref	Ref
Ran out of money, 1 + months in the past year				
Yes	1.68 (1.30–2.17)***	1.62 (1.25–2.11)**	1.52 (1.16–2.00)**	1.36 (1.04–1.77)*
No	Ref	Ref	Ref	Ref
No primary care provider (if HIV + , no HIV PCP)				
Yes	1.19 (0.89–1.60)	1.20 (0.89–1.62)	1.07 (0.79–1.45)	1.13 (0.84–1.51)
No	Ref	Ref	Ref	Ref
Tested for STD in previous year				
Yes	0.72 (0.56–0.94) *	0.72 (0.55–0.95) **	0.68 (0.52–0.90)**	0.74 (0.57–0.97)*
No	Ref	Ref	Ref	Ref
Experienced racism		1.33 (1.15–1.53) ***		1.25 (1.08–1.44)**
Experienced homophobia		1.07 (0.93–1.24)		1.07 (0.93–1.23)
Mental Health and substance use characteristics				
Depressive symptoms		1.02 (0.90–1.13)		0.99 (0.87–1.13)
Resilience		1.05 (0.90–1.21)		1.02 (0.88–1.17)
Binge drank, past 2 months				
Yes			1.19 (0.87–162)	1.08 (0.81–1.45)
No			Ref	Ref
Stimulant use, past 2 months				
Yes			1.33 (0.95–1.85)	1.36 (0.99–1.88)
No			Ref	Ref
Smoking Frequency				
Nonsmoker			Ref	Ref
Non-daily smoker			1.54 (1.12–2.12)**	1.69 (1.25–2.30)***
Daily smoker			2.47 (1.72–3.55)***	2.55 (1.79–3.63)***
Log Likelihood	-782.20	-753.04	-735.44	-782.73
Chi-squared statistic	112.45	142.90	155.57	171.76

^*^p < 0.05

^**^p < 0.01

^***^p < 0.001

In the second adjusted regression model (Table 3), history of incarceration was regressed onto socioeconomic needs, structural discrimination, & mental health, several variables remained significant from Model 1. These variables were: being unsure of their HIV status not having a high school diploma/GED, joblessness, history of homelessness, having run out of money, and history of STD testing. In this model, individuals who identified as bisexual (OR 1.37; 95% CI: 1.08–1.85) and heterosexual/other (OR 3.01; 95% CI: 1.30–6.96) compared to gay/homosexual/same gender loving and those who experienced greater racism (OR 1.33; 95% CI: 1.15–1.53) was associated with a higher likelihood of reporting having a history of incarceration.

In the third adjusted regression model (Table 3), history of incarceration was regressed onto socioeconomic needs, structural discrimination, & substance use, and several variables remained significant from Model 1. These variables were: being unsure of their HIV status, not having a high school diploma, joblessness, having run out of money, and history of STD testing. In this model, individuals who identified as heterosexual/other (OR 2.38; 95% CI: 1.02-5.56) compared to gay/homosexual/same-gender and being a non-daily smoker (OR 1.54 95% CI: 1.12–2.12) or who indicated being a daily smoker (OR 2.47; 95% CI: 1.72–3.55) compared to being a nonsmoker was associated with a higher likelihood of reporting having a history of incarceration.

In the fourth adjusted regression model (Table 3), history of incarceration adjusting was regressed onto socioeconomic needs, structural discrimination, mental health & substance use, HIV status, several variables again remained significant from Model 1. These variables were: not having a high school diploma/GED, joblessness, having run of money, and history of STD testing. In this model, higher level of racism (OR 1.25; 95% CI: 1.08–1.44) and being a non-daily smoker (OR 1.69 95% CI: 1.25–2.30) or daily smoker (OR 2.55; 95% CI: 1.79–3.63) compared to being a nonsmoker were more likely to report a history of incarceration.

## Discussion

This study examined the association between socioeconomic needs, structural discrimination, and history of incarceration among a sample of young Black SMM in Houston and Dallas, Texas. We found that more than a quarter (26%) of the sample reported a lifetime experience of incarceration. Additionally, participants with unmet socioeconomic and structural needs (unemployment, homelessness, financial insecurity and limited educational attainment) were more likely to have a history of incarceration. These findings reinforce the need for intervention development for young Black SMM who are at risk for incarceration, are currently or have been recently incarcerated. Together, these results suggest heightened vulnerability that could result in recidivism and perpetuate a cycle of social and economic disenfranchisement.

We found that history of incarceration was associated with several socioeconomic and structural needs including lack of employment, homelessness, financial insecurity, and lower educational attainment. These findings are in line with a longitudinal study of young Black SMM in Chicago, IL that found that economic hardship, previous criminal justice involvement and substance was associated with incident criminal justice involvement over 18-months of follow-up [30]. These various social and economic positions can be mutually reinforcing such that the possibility for upward mobility becomes almost impossible. For example, not having a high school diploma or GED can limit employment opportunities, potentially compounding the stigma of having been previously incarcerated as a barrier to employment. Furthermore, an individual without stable employment is at higher risk for homelessness, which can also impede employment, a potential pathway to improved economic security. Homelessness can also increase risk for incarceration [31], either due to individuals resorting to survival strategies (e.g., theft, exchange sex), through policies “quality of life” policing (policy of allocating additional law-enforcement resources to areas where crime is believed to be endemic) that arrests homeless persons for trespassing or sleeping on public property. Consequently, it is imperative that interventions are developed to address the basic social and economic needs of young Black SMM with a history of incarceration or who are at risk of incarceration. An intervention that provides resources around housing assistance, cash assistance, food resources, and job placement, and is facilitated by a dedicated case worker experienced in navigating local and state government safety net programs could address these overlapping and potentially self-reinforcing factors. Addressing these basic needs may reduce risk for incarceration or reincarceration among young Black SMM.

Participants who had a history of incarceration were also more likely to report experiences of racism. It is important to note that young Black SMM are situated at the intersection of multiple minoritized identities on the basis on race and sexual orientation [32–35]. Additionally, the racialized public perception of the criminal justice system might put young Black SMM with a history of incarceration at higher risk for experiencing racism. It is important to note that after adjusting for other variables, experiencing of homophobia was not significantly associated with having been incarcerated, providing more evidence about the critical role race plays in the experiences of young Black SMM with a history of incarceration. It is also possible that racist prejudices toward Black men who have been involved with the criminal justice system might be more pervasive than homophobic prejudices associating SMM with criminality. This phenomenon illuminates the need for larger public discourse on the structure and functioning of the criminal justice, especially related to how it is overly punitive toward young Black men [36, 37]. Reform of the criminal justice system, especially as it relates to sentencing laws, bail reform, ‘ban the box’ policies, and juvenile justice reform are desperately needed. Specifically, ‘ban the box’ reform, which posits that employers should first consider a potential job candidate’s qualification, without the stigma of a prior conviction or arrest, could result in more economic opportunities of Black SMM with a history of incarceration. It is important to note that the state of Texas, which is where this study was conducted, does not have a ‘ban the box’ statute at this time.

While depressive symptoms and resilience were initially significantly associated with history of incarceration, those effects became nonsignificant in the multivariable models. This provides further evidence about the need to address the underlying socioeconomic and structural needs, which also have implication for mental health. As we described earlier, there is a need for structural intervention and safety net services to support re-entry for formerly incarcerated young Black SMM, both socially and economically. Social cognitive and behavioral interventions are individualistic (implicitly locating a deficit within the individual and their behavior) and in isolation are insufficient for addressing these socioeconomic needs. One such model is the Louisiana Integrated Center for Care, Supportive Services, and Community Health recently described by Brewer et al. [18]Within the demonstration project, that brought together a range of stakeholders and service providers to address social and structural drivers of HIV disparities among black men, three priority areas were: 1) reducing barriers to HIV care during re-entry for incarceration-involved young black men, 2) addressing socioeconomic disparities among young Black SMM and 3) an assessment of and communications campaign to address housing discrimination experienced by young Black SMM[18].

Our study had some limitations. First, the self-reported nature of the data could have resulted in social desirability bias, and possibly due to fear of experiencing stigma and discrimination, especially for participants with a history of incarceration, who experience societal-level prejudice. This bias may have resulted in an underestimation of prevalence of incarceration history and other pertinent variables. Second, we did not collect data on the reason for incarceration, length of time, or number of times incarcerated. In a similar sample of young Black SMM, reasons for incarceration included substance use or substance dealing, intimate partner violence, physical altercations, sex work, and financial fraud)[19]. Consequently, there could be different antecedents or outcomes associated with different reasons for incarceration that is not captured in our data or analysis. Next, it is plausible that these independent variables (structural and socioeconomic needs) are antecedents of incarceration, and/or outcomes of incarceration. Existing longitudinal datasets, including those with an original focus on HIV outcomes, may have captured enough socioeconomic indicators to support a new, secondary analysis that sheds more light on the temporality of these relationships and most critical points for intervention. Our present findings can provide insight into relevant factors to incorporate. Finally, our data do not include the number of incarcerations experienced, the length of these incarceration experiences, the setting of incarceration (e.g., jail versus prison) or the recency of incarceration. This contextual information could be addressed in future primary data collection or secondary data analysis (e.g., the differential effects of multiple incarcerations).

## Conclusions

A vast majority of research examining incarceration among young Black SMM have been related to HIV disparities, thereby telling an incomplete story. Housing instability, unemployment, and limited educational advancement are structural inequities found to be associated with incarceration among young Black SMM. Currently, programs are warranted that address these factors as part of a more holistic response to the social and structural disadvantage that young Black SMM navigate daily. To date, there are few, if any, re-entry programs for formerly incarcerated young Black SMM that address housing, education, and employment needs, and they are desperately needed. Future research should: 1) examine the mechanisms and pathways underlying the associations between socioeconomic needs and incarceration among young Black SMM (as antecedents, outcomes, and both), 2) test the cultural appropriateness and relevance of existing re-entry programs for the general population, and whether these programs adequately reach young Black SMM or need tailoring, and 3) investigate whether experiences of racism may differ for young Black SMM who have experienced incarceration versus those who have not (e.g., frequency, sources, perceived impacts), to shed further light on the most potent sources of disadvantage and discrimination that exacerbate incarceration disparities and incarceration sequalae for young Black SMM .

## Data Availability

Data is available upon request.
